# A New Beginning: Young Women’s Experiences and Sexual Function 18 Months After Bariatric Surgery

**DOI:** 10.1016/j.esxm.2020.08.007

**Published:** 2020-09-24

**Authors:** Emma Nilsson-Condori, Stina Järvholm, Ann Thurin-Kjellberg, Jan Hedenbro, Britt Friberg

**Affiliations:** 1 Division of Reproductive Medicine, Department of Translational Medicine, Lund University, Lund, Sweden; 2 Department of Obstetrics and Gynecology, Institute of Clinical Sciences, Sahlgrenska Academy, Gothenburg University, Gothenburg, Sweden; 3 Division of Reproductive Medicine, Sahlgrenska University Hospital, Gothenburg, Sweden; 4 Department of Surgery, Clinical Sciences, Lund University, Lund, Sweden

**Keywords:** Sexual Function, Bariatric Surgery, Female Fertility, Obesity

## Abstract

**Introduction:**

Female patients expect improved quality of life, including sexual health and regain of fertility after bariatric surgery. Little has been published on to the extent to which patients’ expectations are met by the weight loss after surgery.

**Aim:**

To explore how women perceive the effects of bariatric surgery on quality of life, focusing on sexual health and fertility.

**Methods:**

A qualitative study based on thematic analysis, supported by questionnaire data. Interviews following a semistructured guide were conducted with childless women (n = 11) aged 25–34 years recruited from a university-affiliated Swedish bariatric center. The interviews took place 18 months after surgery in the participants’ homes or at the hospital and were recorded and transcribed verbatim. Data were analyzed with a thematic approach. Questionnaires were filled in at the time of the interviews and compared with preoperative data using a Wilcoxon test for paired data.

**Main outcome measures:**

Participants described experiences related to female sexual health after bariatric surgery. The Hospital Anxiety and Depression Scale and the Female Sexual Function Index questionnaires were administered preoperatively and postoperatively.

**Results:**

“A new beginning” was identified as the master theme, with 3 underlying subthemes: “Being worthy of love,” “Exploring sexuality,” and “Considering parenthood.” The participants described a transformation into being more comfortable with themselves that affected all areas of life, including sexual life. These findings were supported by lower scores for depression, 6.5 vs 2, and improved total Female Sexual Function Index scores, median 23.3 preoperatively and 29.1 postoperatively, *P* = .012.

**Conclusions:**

Improved body image and enhanced self-esteem play important roles in improved sexual functioning in women after bariatric surgery.

**Nilsson-Condori E, Järvholm S, Thurin-Kjellberg A, et al. A New Beginning: Young Women’s Experiences and Sexual Function 18 Months After Bariatric Surgery. Sex Med 2020;8:730–739.**

## Introduction

The prevalence of obesity, defined as a body mass index (BMI) of >30, has nearly tripled worldwide since 1975.^1^ In Sweden, data from 2018 show that 7% of women aged 16–29 years are obese, and the number has increased to 14% in the age group 30–44 years.^2^ Obesity is associated with female infertility and a doubled time to pregnancy.^3^ Bariatric surgery is the most effective treatment for obesity, and the majority of treatment-seeking patients are women, 75% of those being of fertile age in accordance with national Swedish data.^4^^,^^5^

However, young women are generally a vulnerable group. National statistics from 2018 showed self-reported mental distress in 33% of women aged 16–29 years.^6^ Bariatric surgery candidates, as well as young obese adults seeking behavioral weight reduction treatment, have been shown to have a high prevalence of depressive and anxiety disorders.^7^^,^^8^

The perception of the body represents an integral part of self-image and is important to consider in relation to perceived stress and internalized problems among adolescent girls and young women.^9^^,^^10^ Related to negative stress, the body can be viewed as both a source of, and site for, mental stress. Idealized body images and appearance-related pressure are found to be major sources of stress in girls.^11^^,^^12^

A poor evaluation of and behavior toward body image is detrimental to women’s sexual functioning, and dissatisfaction with one’s body has been found to predict decreases in desire and arousal.^13^ Approximately one half of women before bariatric surgery are dissatisfied with their sexual life as compared with 16% among sexually active Swedish women aged 18–65 years.^14^^,^^15^ Several large-scale survey studies have demonstrated that sexual satisfaction in women is associated with emotional well-being, partner satisfaction, and overall quality of life.^16^ Female sexual dysfunction improves after bariatric surgery.^17^^,^^18^ There is also some evidence that the incidence of pregnancies increases after bariatric surgery, but the mechanisms behind this effect remain unknown.^19^^,^^20^ In a previous qualitative study, young childless women had high expectations on getting back to a normal life including better sexual health and improved fertility.^21^

There is not much literature about young women, their identity, self-image, and sexual health after bariatric surgery. The aim of this qualitative study was to explore how women perceive the effects of bariatric surgery on quality of life, focusing on sexual health and fertility.

## Materials and methods

### Recruitment

**Figure 1:**
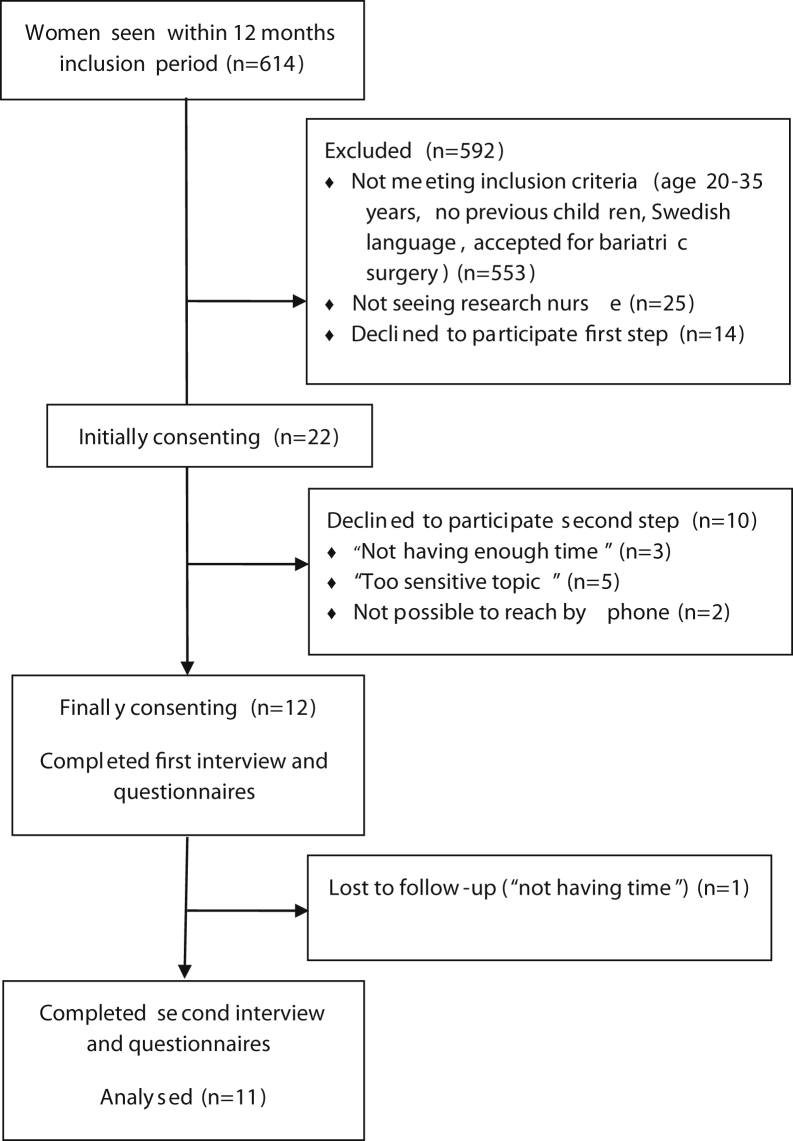
Flow diagram inclusion participants.

From a single bariatric center in Malmö, Sweden, between April 2016 and April 2017, Swedish-speaking women without previous children, aged 20–35 years, and who had been accepted for bariatric surgery (both public and privately funded) were identified and consecutively invited to the study, see Figure 1. Written informed consent was obtained from all participants. The study was approved by the Ethics Review Board in Lund, # 2016/50, and conducted in accordance with the Helsinki Declaration.

For this follow-up study, 18 months after surgery, the women were contacted individually by telephone by the first author (ENC) as previously agreed after the first interview. 11 of the initial 12 women agreed to participate in the follow-up, 1 woman declined referring to not having time because of a heavy workload, see Figure 1.

### Data Collection

Interviews were conducted once with each participant by ENC, either at the hospital (3 of 11) or if the participants so preferred, in their own home (8 of 11). In 4 cases, a partner was present in the home but did not take part in the interview. Before the interview, the participants completed the Hospital Anxiety and Depression Scale (HADS) and the Female Sexual Function Index (FSFI) questionnaires. The participants had previously received written information about the study and the researcher. Before starting the interview, they were reminded of the purpose of the research and that it would not be a medical consultation.

The semistructured interview guide, developed by ENC and SJ (the second author), covered the following topics: health after bariatric surgery including self-reported weight loss, psychological aspects of reproduction, fertility, expectations of surgery and future fertility, and information, see Supplemental Material 1. The interviews were 25–86 minutes long (median 38 minutes). All interviews were audio recorded and transcribed verbatim. Short field notes were taken regarding the setting of the interview.

The FSFI is a multidimensional self-report instrument for assessing important aspects of sexual function in women, where higher scoring indicates better function/less pain.^22^ The HADS is a 14-item self-report screening scale that consists of a 7-item anxiety subscale and a 7-item depression subscale, the scale performs well in assessing the symptom severity and caseness of anxiety disorders and depression in the general population.^23^ For practical reasons, body weight, height, and BMI were all measured at baseline and at the 1-year follow-up at the bariatric center.

### Data Analyses

**Figure 2:**
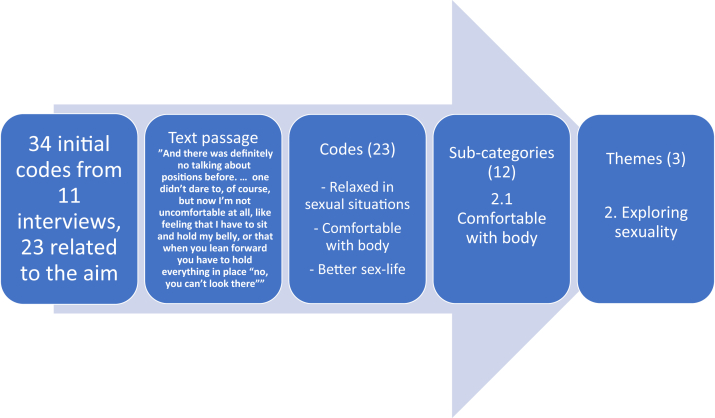
The data analysis; example of coding organized into subcategories and main themes.

The interview material was analyzed inductively using thematic analysis, in accordance with the methods of Braun and Clarke.^24^ This method was chosen because the approach was explorative, with the aim of increasing knowledge about individual experiences. First, the authors (ENC and SJ) individually familiarized themselves with the data by reading the interviews, and then, they noted their first impressions. The analyses were then made by ENC, a female medical doctor working clinically at a fertility center with previous experience of female patients with overweight and obesity, and by SJ, a female psychologist working clinically in another fertility center and with previous experience of qualitative research. None of the authors had previous personal experience of bariatric surgery or obesity. ATLAS.ti 8.2.34 (Atlas.ti, Berlin, Germany) was used to facilitate the manifest analysis. The first author created 34 diverse initial codes derived from the interview material where 23 were related to the aim, see an example of the coding in Figure 2. Because this was a follow-up study, the number of participants was already fixed, but it is notable, regarding data saturation, that no new codes were created after the seventh interview. Thereafter, ENC and SJ organized the codes into 4 broad themes: (i) Worthy of love, (ii) Find love, (iii) Explore sexuality, and (iv) Maybe a parent, with a total of 17 underlying categories. In the next stage of the analysis, the 4 themes were restructured into 3 main themes: (i) Being worthy of love, (ii) Exploring sexuality, and (iii) Considering parenthood. In total, these 3 had 12 subcategories.

After this, ENC and SJ worked together on the model and the authors agreed on a final understanding of the themes and sub-categories shown in Table 3.

Anthropometric and questionnaire data were stored in a proprietary database with access only for the authors. Quantitative data were analyzed using the Wilcoxon test for paired data, 2-tailed *P*-values were used, and the level of statistical significance was set at *P* < .05. Analyses were performed using IBM SPSS Statistics, version 24 (IBM Corp, Armonk, NY).

## Results

The mean age of the 11 participants was 28.8 years, 10 of the participants had undergone laparoscopic gastric bypass surgery and 1 participant had had a laparoscopic gastric sleeve surgery. Weight and BMI reductions were significant (*P* = .008), as was the reduction in waist circumference (*P* = .028) (Table 1). None of the participants had undergone plastic surgery. At the time of the follow-up, 10 of 11 participants were in a relationship, and 7 had a new partner, see Table 2. One participant was pregnant, and another had already delivered a healthy baby.

**Table 1:** Anthropometric data at baseline and 13 months after operation

Anthropometric data median (range)	Preoperatively	Postoperatively	*P*-value
Body weight, kg	117 (84–148)	83 (55–90)	.008
BMI, kg/m^2^	41.9 (32.8–49.5)	29.1 (22.0–33.2)	.008
Waist circumference, cm	118 (96–135)	92 (68–104)	.028

BMI = body mass index.

**Table 2:** Participants' characteristics

Variable	Baseline	Postoperatively
No.	12	11
Age median (range)	27 (23–32)	29 (25–34)
Ethnicity		
Swedish ancestry	9	8
Other	3	3
Highest level of education		
University	3	3
Secondary School	9	8
Occupation		
Employed	6	9
Unemployed	4	2
Student	2	0
In a romantic relationship	5	10
New partner	0	7
Menstrual irregularities	7	0

**Table 3:** The data analysis; master theme, main themes, and underlying categories

Master theme: a new beginning	
1. Being worthy of love	1.1 Reflected appraisal
	1.2 Finding myself
	1.3 Active and outgoing
	1.4 Finding a partner
	1.5 Continuous body improvement
2. Exploring sexuality	2.1 Comfortable with the body
	2.2 Daring to make demands
	2.3 Improved sexual functioning
	2.4 Lacking desire
3. Considering parenthood	3.1 The body seems ready
	3.2 Planning for children
	3.3 The uncertain fertility

A New Beginning was identified as the master theme, affecting the 3 underlying main themes, see Table 3.

### Being Worthy of Love

The participants described how they were now much more satisfied with their own body and appearance. Self-esteem felt improved, and inhibitions were lowered. The majority were in a romantic relationship (10 of 11).

#### Reflected Appraisal

Several of the participants said that they no longer had thoughts of what other people might think of them and their body. This had previously restricted them in several everyday areas of their lives. Reflections about how it might have been all in their own head before came up, but nonetheless they now felt liberated from these worries.

*“Now, I no longer feel like it’s uncomfortable to go exercising among other people, like they’d be thinking “what is she doing” and so on. I can do that. I am much, much more comfortable in social contexts, like I said. Going to birthday parties and, like hanging out with friends and so on, that feels great too.”* Participant 10

#### Finding Myself

The participants described the process of finding themselves again. Some of them referred to the normal-weight person they had been before, which seemed easier than for those who never had a normal-weight version of themselves before.

*“It’s more that I’ve come back to what I****should****be like. Then I didn’t get this, like others did: “oh, I don’t think that I look slim” but I instead start to recognize myself again, like, “hey, this is me”. So, it’s a bit different to me.”* Participant 4

The informants who never before had been normal-weight talked about still being “the fat one” in their own head.

*“I guess that deep inside I’m always gonna be the fat one.”* Participant 1

#### Continuous Improvement

Except for 1, all participants were very content with their weight loss, but none of them was content with the body. The body was described as an object that needed continuous improvement. Some felt troubled by excess skin or the loss of breast mass, both issues leading to a perceived need for plastic surgery.

*“I have to tone the belly there” and then “I’ve gotta buy some boobs.” Greedy, one could say that I am, or you just want to get better and better. I think now, it’s not about…, if you think about when you were younger it was all about being thin and slender, sort of. But now I want, now it’s the muscles that I’m looking for and so on….”* Participant 1

Others focused on excessive weight loss that had changed body proportions, or other perceived problematic areas of the body.

#### Active and Outgoing

Life had changed; as the body was lighter, things in everyday life got easier. This facilitated exercising but also accepting invitations for social events, as well as new activities involving physical activity—making the participants more outgoing.

*“I feel much better, I’m more comfortable. Like, I can be with other people. That’s fine. It doesn’t feel awkward anymore.”* Participant 5

#### Finding a Partner

10 of 11 participants were now in a relationship. Shortly after surgery, several of them had met a (new) partner, and the ones that had already been in a stable relationship before surgery had got married or were planning for marriage.

*“Definitely. I’ve found my other half now. I have. So that’s a lot. He’s comfortable with me, and I’m comfortable with him. He’s not judging. You can notice that he likes me and the way I look.”* Participant 8

### Exploring Sexuality

Most of the participants described a more active sex life, which was also more satisfying than before surgery. Internal factors, such as being more comfortable in a sexual situation and enhanced self-esteem, allowed them to demand more of their partners.

#### Comfortable With the Body

Being comfortable with the body and being naked, led to more intimacy. Sex was also a lot more enjoyable when one was able to let go of the thoughts of how one’s own body might appear.

*“And there was definitely no talking about positions before. … one didn’t dare to, of course, but now I’m not uncomfortable at all, like feeling that I have to sit and hold my belly, or that when you lean forward you have to hold everything in place “- No, you can’t look there””* Participant 8

#### Daring to Make Demands

The enhanced self-esteem made it easier to make demands. The participants talked about feeling relaxed about guiding the partner to better sex, and the stimulation needed to reach climax.

*“… and then not being afraid of saying what you want and so on. So just, … really, to be comfortable with yourself leads to a thousand other things around sex that makes it a much, much better experience and makes it more pleasant, and makes it, like, easier to have orgasms.”* Participant 10

#### Improved Sexual Functioning

Several participants described increased desire and a more active sex life. They pointed out factors such as increased energy levels and endurance, which made having sex more interesting.

Another participant pointed out less need of lubricants as the cycle had become more regular and ovulatory. Yet another said that the weight loss had increased “the feeling of having intercourse.”

*“I had some difficulties getting wet before … since my ovulations started again, we haven’t needed much lubricant at all.”* Participant 4

#### Lacking Desire

4 of the participants talked about not having much sexual desire. These women also described anxiety and depressive symptoms, and 3 of them were taking antidepressants, which they thought might be the cause. The fourth woman was inhibited by pain from worsened endometriosis.

*“I…, well since…, because I’ve got this depression I’m not in the mood for intimacy…, that is neither intercourse nor closeness at all, really. It feels like I’m rejecting him, which I don’t want him to…, I mean, to feel. So, in that way it’s worsening the entire situation, so to say.”* Participant 3

### Considering Parenthood

One of the participants had already become a parent, and a second was pregnant. The other participants said that they wanted to have children in the future, but not all of them felt ready to get pregnant yet. Having regular cycles was considered very positive, as a marker of female fertility.

#### The Body is Ready

Most of the participants now had a regular cycle and expressed their joy at feeling like a normal woman again. They talked about feeling relieved as the body was working as it was supposed to again. For the participants who wanted to conceive now, ovulation was very important.

*“And people are complaining about their…., I love my period.”* Participant 4

#### Planning for Children

Some of the participants had gone through surgery to enhance their fertility and were now trying to conceive. Another 2 had already got pregnant when they had met a partner. All participants were planning for children in the future.

*“I hope…, so before…, I haven’t got pregnant before, but I was rather thinking that I can’t get pregnant, but I’m still hoping that I actually could. I really would like to have a family at some point.”* Participant 3

When talking about not being ready to get pregnant, other factors than weight loss were described as important, such as getting to know the partner better or having stable economic circumstances.

*“No, not really, since he was not done with his studies and didn’t have a permanent job, err, so we never got that far. We didn’t. Like I told you, these are priorities that you want to be done with before having a family.”* Participant 1

Several of the participants planned to postpone pregnancy until 2 years after surgery, on the advice of healthcare staff.

#### The Uncertain Fertility

Although most of them now had a regular cycle, several talked about feeling stressed about fertility and said they still did not feel certain they would conceive when they felt ready. Some had friends who had experience of infertility, while others referred to their own previous difficulties conceiving.

“I: And now you’re thinking more about it (having children) then?

*R: Yes, actually. Then this with…, yes, but since we tried before to achieve a pregnancy, and I didn’t conceive and so on. Probably I’ve got difficulties with that, I mean.”* Participant 6

### Mood

Scores for depression 18 months postoperatively were significantly lower than those preoperatively, 6.5 vs 2, *P* = .007. Preoperatively, 6 of 12 participants scored 8 or higher as in doubtful regarding depression, but postoperatively, no participant scored more than 7 (Normal). Scores for anxiety were lower postoperatively, 10 (doubtful) vs 7 (normal) although not significant *P* = .137. Preoperatively, 7 of 12 participants scored 8 or higher (doubtful or clinical problems) for anxiety, and postoperatively, 5 of 11 participants scored 8 or higher as seen in Table 4.

**Table 4:** Comparison of questionnaire data preoperatively vs 18 months postoperatively

Variable	N		Median	Minimum	Maximum	*P*
	Valid	Missing				
HADS						
Anxiety preoperatively	12	0	10,0	4,0	16,0	.137
Anxiety postoperatively	11	1	7,0	2,0	14,0	
Depression preoperatively	12	0	6,5	3,0	13,0	.007
Depressions postoperatively	11	1	2,0	0,0	7,0	
FSFI						
Desire preoperatively	11	1	2,4	1,2	4,8	.014
Desire postoperatively	11	1	3,6	2,4	6,0	
Arousal preoperatively	11	1	4,5	0,0	5,7	.018
Arousal postoperatively	11	1	5,4	3,3	6,0	
Lubrication preoperatively	11	1	4,8	0,0	6,0	.028
Lubrication postoperatively	11	1	5,4	3,3	6,0	
Orgasm preoperatively	11	1	4,8	0,0	6,0	.106
Orgasm postoperatively	11	1	4,4	1,6	6,0	
Satisfaction preoperatively	11	1	2,8	1,2	6,0	.025
Satisfaction postoperatively	11	1	5,2	3,2	6,0	
Pain preoperatively	11	1	3,6	0,0	6,0	.027
Pain postoperatively	11	1	6,0	1,2	6,0	
FSFI total preoperatively	11	1	23,3	4,2	34,2	.012
FSFI total postoperatively	11	1	29,1	20,5	36,0	

FSFI = Female Sexual Function Index; HADS = Hospital Anxiety and Depression Scale.

### Female Sexual Functioning

**Figure 3:**
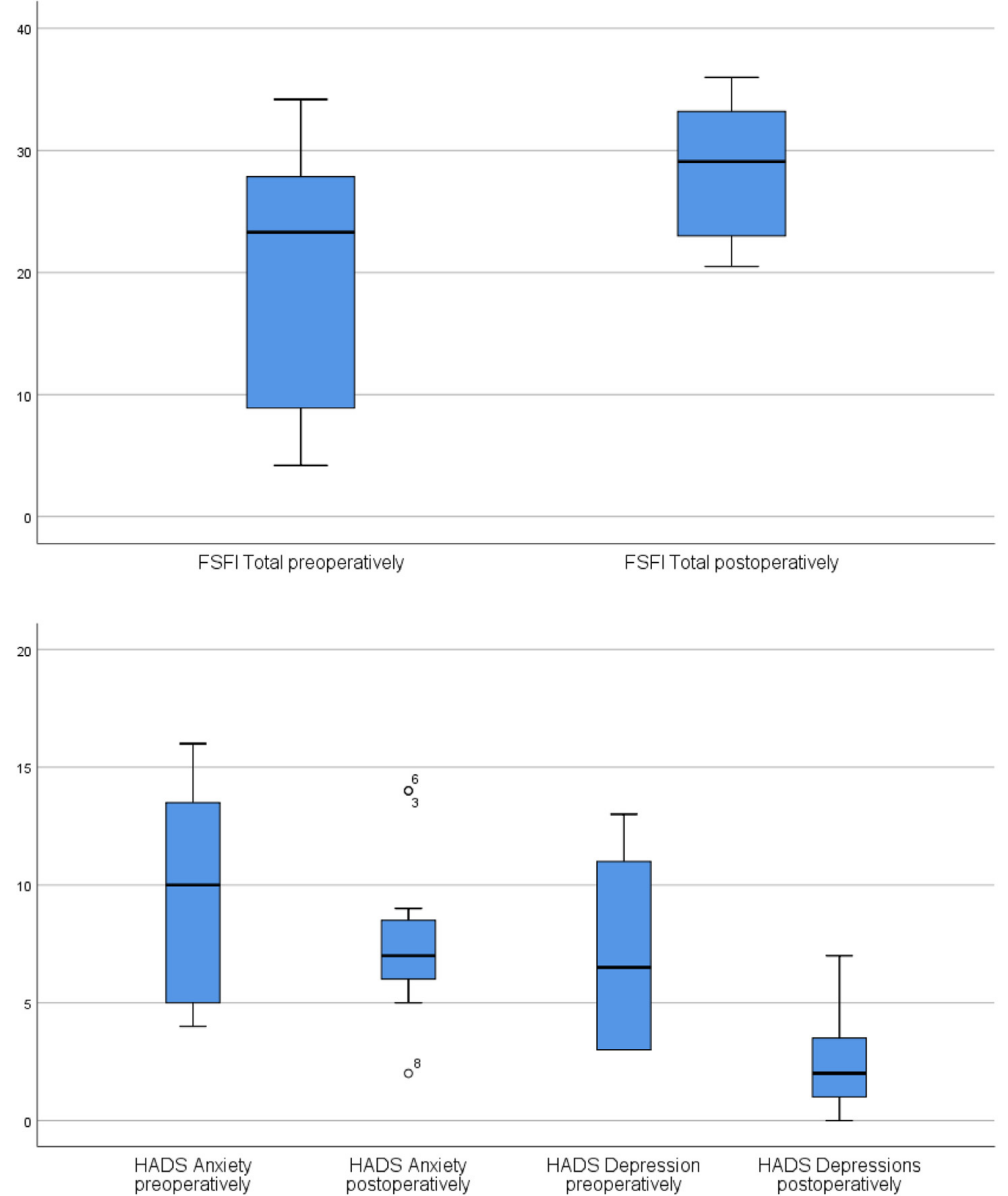
FSFI total scores and HADS scores for anxiety and depression. FSFI = Female Sexual Function Index; HADS = Hospital Anxiety and Depression Scale.

The total FSFI score was significantly improved from a median of 23.3–29.1, *P* = .012. The participants scored significantly higher on most domains of the FSFI, except for orgasm, where there was no significant difference, see Table 4 and Figure 3.

## Discussion

Obesity is a common disease, and bariatric surgery is the only therapeutic modality to give profound and long-lasting weight loss. Because young women now are seeking bariatric surgery, we need information on the expected outcome on factors other than body weight, diabetes resolution, and other well-studied variables. Little has been published on to the extent to which patients’ expectations are met by the weight loss after surgery. This study focuses on quality of life regarding sexual health and patients’ considerations about fertility/pregnancy.

After bariatric surgery, the women described how weight reduction had enhanced their self-esteem, affecting several areas of life, and how it had also improved their sexual life. The interview data and the significant results of questionnaire data from the FSFI and HADS pointed in the same direction.

The master theme *A New Beginning* represents the optimistic views on changes that had already taken place or that were hoped for, in the near future. The changes were related to self-image regarding all the 3 subthemes *Being worthy of love, Exploring sexuality* and *Considering parenthood*. Similarly, in another qualitative study by Bocchieri et al^25^ identified patients reported a rebirth/transformation after bariatric surgery.

The main theme *Being worthy of love* reflects how the changed body image affects well-being and self-esteem in relation to social interactions. The physical body is important to identity in both positive and negative ways.^9^^,^^10^^,^^26^ Jensen et al^27^ suggested that perception of control may be an essential aspect of body image and the key to understanding these young women’s feelings of empowerment and quality of life through body control after bariatric surgery. Being *Comfortable with the body* made our participants relax in a sexual context. Body shame has previously been shown to predict greater self-consciousness during physical intimacy, and the relationship between body shame and sexual pleasure and problems is mediated by sexual self-consciousness.^28^

Parenthood could be viewed as a central goal in the self-image in our group of young women. The participants expressed their joy at regaining regular cycles as a marker of fertility. It is recommended to delay pregnancy by 12–18 months after bariatric surgery. The unplanned pregnancies were welcomed by both of the women who had conceived, but could also be seen as in line with previous findings of a link between BMI, sexual behavior, and adverse sexual health outcomes, with more unplanned pregnancies.^29^

Sexual function was significantly improved, in accordance with several previous studies on sexual functioning after bariatric surgery.^17^^,^^18^^,^^30^ Improvements in sexual life after bariatric surgery have previously been linked to a decrease in BMI and also linked to decreased body image dissatisfaction.^31^ In a study on women with polycystic ovary syndrome and normal, overweight, and obese BMI, there was a negative correlation between HADS-A and HADS-D and the FSFI total score, suggesting a link between body dissatisfaction, distorted self-perceived body image, sexual dysfunction, and depression.^32^ In our study, participants scored significantly lower postoperatively on HADS-D and highlighted the increased self-esteem as crucial for increased satisfaction with their sexual life. More participants were also partnered at follow-up, which in part could mediate the improvement in the FSFI.

We did ask the participants for suggestions on additional care but did not find any emerging theme on this topic. Our participants were content with their decision to undergo surgery, a choice they had all made themselves. It was notable that the positive narrations sometimes sounded as if they had been cut out of an advertisement brochure. This could possibly be explained by surgery being seen as successful when previous expectations were reached, as described by da Silva and Maia Ada^33^ Although several studies have shown improvement in sexual functioning after bariatric surgery, long-term follow-up has also shown that the effect diminishes with time, and our participants were interviewed 18 months after surgery.^18^

### Limitations

The main strength of the present study is the qualitative design with prolonged engagement and the low level of loss to follow-up. The participants represent the Swedish demographics well. Another strength is the use of the validated FSFI and HADS questionnaires that were subordinate to the qualitative data but supported with quantitative data. The main limitation is the selection bias related to the patients that agreed to participate in the study. Self-selection may bias participants who are open minded regarding questions about sexuality. The participants were encouraged to speak freely about their experiences, but it is necessary to reflect on whether patients after bariatric surgery may be more influenced by social desirability in their answers than other groups. It must also be taken into account that this study was limited by its sample size, and also by the form of recruitment of patients from a single bariatric center in the south of Sweden, and therefore may not be representative of all bariatric patients. Our results need to be confirmed in long-term quantitative studies including large cohorts of patients and controls.

## Conclusion

As expressed by the participants in our study, improved body image and self-esteem were important to facilitate finding a partner and also played important roles in improving sexual functioning. This was confirmed by improved FSFI scores and lower levels of depression. Our findings are of importance when addressing future healthcare needs in this rapidly expanding group of patients.

## Statement of authorship

Emma Nilsson-Condori: Conceptualization, Methodology, Investigation, Project Administration, Formal Analysis, Writing - Original Draft, Writing Review & Editing; Stina Järvholm: Conceptualization, Methodology, Investigation, Formal Analysis, Writing - Review & Editing; Ann Thurin-Kjellberg: Conceptualization, Methodology, Resources, Funding Acquisition, Writing - Review & Editing; Jan Hedenbro: Conceptualization, Methodology, Investigation, Formal Analysis, Resources, Writing - Review & Editing; Britt Friberg: Conceptualization, Methodology, Resources, Writing - Review & Editing, Funding Acquisition, Supervision.
