# Supplementary material for: A New Beginning: Young Women’s Experiences and Sexual Function 18 Months After Bariatric Surgery
Source: Sex Med. 2020 Sep 24;8(4):730–9. doi: 10.1016/j.esxm.2020.08.007 (PMC7691881; doi:10.1016/j.esxm.2020.08.007)
Supplement: Supplemental Material 1 [file mmc1.doc]

# INTERVIEWGUIDE women after bariatric surgery

Introduction of background of the research project

## Can you please describe why we are here today?

HEALTH AFTER BARIATRIC SURGERY

How is your physical health?

How is your psychological health?

Have you got any other health issues?

Are you menstruating?

Are you content with your body?

Is there today anything you avoid doing because of your weight?

Do you feel comfortable in the company of other people?

PSYCHOLOGICAL ASPECTS ON REPRODUCTION

Are you in a relationship right now?

How long have you been in this relationship?

Have you had previous relationships? How have they been?

How is your sexual life?

How is your libido?

How do you experience your partners libido?

Have you ever, during life, considered becoming a parent?

FERTILITY

Have you previously been pregnant?

(What happened?)

(What are your thoughts on this?)

Have you previously tried to get pregnant?

Do you want to get pregnant?

(Do you remember the first time you wanted to be a mother?)

(What happened when you tried to get pregnant?)

EXPECTATIONS ON SURGERY AND FUTURE FERTILITY

What were you hoping that would change after the surgery?

Did you have any fears related to go through the surgery and life afterwards? Were your fears realized?

What do you think about the effects of the surgery on relationships?

Do you think the surgery has affected your sexual life? Has it become better or worse?

What do you think about the effects of the surgery on the possibility to get pregnant?

What do you think about the effects of the surgery on a possible future pregnancy?

INFORMATION

Have you got any information about childbearing after the surgery?

What is your opinion about the information from health care in these matters?

Have you been searching the internet on your own for information in these matters?

Have you found any information about this in other media, such as radio, tv, papers?

Has health care (staff) affected your decision to go through surgery?

OTHER

Is there anything else about this topic that you would like to add, or anything about childbearing that we have not talked about that you would like to tell?
